# A Modified Mediterranean Ketogenic Diet reverses signatures and mitigates modifiable risk factors of Alzheimer’s disease

**DOI:** 10.1002/alz.089878

**Published:** 2025-01-09

**Authors:** Annalise Schweickart, Kevin Huynh, Richa Batra, Bryan J. Neth, Cameron Martino, Amanda H. Dilmore, Corey Giles, Tingting Wang, Natalie A Mellett, Thy Duong, Liat Shenhav, Anru Zhang, Pixu Shi, Naama Karu, Kiana West, Jasmine Zemlin, Gibraan Rahman, Morgan Panitchpakdi, Michael Meehan, Kelly C Weldon, Thomas C. Register, Matthias Arnold, Peter J Meikle, Leyla Schimmel, Kaj Blennow, Henrik Zetterberg, Colette Blach, Pieter Dorrestein, Rob Knight, Jan Krumsiek, Rima Kaddurah‐Daouk, Alzheimer’s Gut Microbiome Project Consortium (AGMP), Suzanne Craft

**Affiliations:** ^1^ Institute for Computational Biomedicine, Englander Institute for Precision Medicine, Department of Physiology and Biophysics, Weill Cornell Medicine, New York, NY USA; ^2^ Baker Heart and Diabetes Institute, Melbourne, VIC Australia; ^3^ Mayo Clinic, Rochester, MN USA; ^4^ University of California, San Diego, La Jolla, CA USA; ^5^ New York University Grossman School of Medicine, New York City, NY USA; ^6^ Duke University, Durham, NC USA; ^7^ Tasmanian Independent Metabolomics and Analytical Chemistry Solutions, Hobart, TAS Australia; ^8^ Wake Forest University School of Medicine, Winston‐Salem, NC USA; ^9^ Helmholtz Zentrum Muenchen, Oberschleißheim Germany; ^10^ University of Gothenburg, Mölndal Sweden; ^11^ University of Gothenburg, Mölndal, Gothenburg Sweden

## Abstract

**Background:**

Alzheimer’s disease (AD) is a neurodegenerative disorder with significant environmental factors, including diet, that influence its onset and progression. While the ketogenic diet (KD) holds promise in reducing metabolic risks and potentially affecting AD progression, only a few studies have explored the KD’s molecular impact for markers of AD therapeutic potential. The BEAM diet study simultaneously profiled the KD’s effect on the lipidome, blood and cerebrospinal metabolome, and microbiome of both cognitively impaired and cognitively normal individuals. The findings summarized here assess the biological impact of a Modified Mediterranean KD in the context of Alzheimer’s disease treatment and prevention.

**Method:**

BEAM involved participants at risk for AD, either cognitively normal or with mild cognitive impairment. The participants consumed both a modified Mediterranean‐ketogenic diet (MMKD) and the American Heart Association diet (AHAD) for 6 weeks each, separated by a 6‐week washout period. We employed HPLC‐MS/MS lipidomics profiling in plasma, nuclear magnetic resonance (NMR)‐based metabolomics to profile serum and CSF, and metagenomics profiling on fecal samples before and after each diet to assess dietary‐induced changes.

**Result:**

The MMKD led to significant alterations in the blood, CSF, and microbiome. These changes included a global elevation across all plasmanyl and plasmenyl ether lipid species, improved modifiable risk factors, like increased HDL‐C and reduced BMI, the reversal of serum metabolic disturbances linked to AD such as an increase in valine levels, and a reduction in systemic inflammation. Leveraging prior clinical studies on AD (n = 1,912), we found that MMKD was inversely associated with the peripheral lipidomic signature of prevalent and incident AD. In the CSF, the MMKD was linked to modified amino acid levels and the breakdown of branched‐chain amino acids (BCAAs). Importantly, we observed a strong correlation between metabolic changes in the CSF and serum, suggesting a systemic regulation of metabolism. In addition, participants with MCI on the MMKD had lower levels of GABA‐producing microbes and GABA, and higher levels of GABA‐regulating microbes.

**Conclusion:**

Our findings highlight that MMKD can improve AD‐related risk factors, reverse some metabolic disturbances associated with AD, and align metabolic changes across the blood‐CSF barrier.

**Funding**: Alzheimer’s Gut Microbiome Project, NIA U19AG063744

